# Virulence Profiles and Fungicide-Target-Gene Variability in *Puccinia graminis* f. sp. *tritici* Populations from Russian Regions

**DOI:** 10.3390/jof12080604

**Published:** 2026-08-13

**Authors:** Ksenia Dudnikova, Olga Baranova, Andrey Shingaliev, Luyen Quoc Lap, Ekaterina Polkhovskaya, Ilya Kirov, Maxim Dudnikov

**Affiliations:** 1 All-Russia Research Institute of Agricultural Biotechnology (FSBSI ARRIAB), 127550 Moscow, Russia; saenkok1997@yandex.ru (K.D.); baranova_oa@mail.ru (O.B.); kronstein491@yandex.ru (A.S.); lapluyenquoc@gmail.com (L.Q.L.); eynzeynkreyn@gmail.com (E.P.); kirovez@gmail.com (I.K.); 2 All-Russian Institute of Plant Protection (FSBSI VIZR), 196608 Saint Petersburg, Russia

**Keywords:** cereal pathogens, *Puccinia graminis* f. sp. *tritici*, stem rust, molecular diagnostics, virulence, fungal genes, SNP, fungicide-target genes, nanopore sequencing

## Abstract

Wheat stem rust, caused by *Puccinia graminis* f. sp. *tritici* (*Pgt*), threatens food security owing to the pathogen’s high virulence and evolutionary variability, as well as the risk of selecting fungicide-resistant forms. For the first time in Russia, we performed a comparative analysis of virulence and genetic diversity in fungicide-target genes (*SdhA–SdhD*, *Cyp51*) in 35 monopustular *Pgt* isolates collected from regions with contrasting fungicide pressure (Saratov, *n* = 12; Chelyabinsk, *n* = 12; Moscow, *n* = 11). Phenotyping on an extended set of nearly isogenic lines carrying different *Sr* genes revealed regional specificity in effective resistance genes (*Sr13*, *Sr26*, *Sr31*, *Sr32*, *Sr35*), underscoring the vulnerability of genetic protection in Russian commercial cultivars. Targeted amplicon sequencing using the Oxford Nanopore Technologies platform produced 175 amplicons (~38,290 long reads, N50 = 1.06 kb), identifying 87% synonymous SNPs, 12% non-synonymous substitutions, and a single nonsense mutation in *SdhA* (position 580). The *Cyp51* and *SdhA* genes were the most polymorphic. Recurrent non-synonymous mutations in these genes were detected in *Pgt* clones from regions with intensive agriculture and high use of chemical plant protection products. These results support the need for predictive breeding of resistant cultivars and adaptation of chemical control within integrated *Pgt* management in the European part of Russia.

## 1. Introduction

In grain crop agroecosystems, fungal pathogens cause significant yield losses, reaching 21% of global production [1], underscoring their role in threatening food security. Among these pathogens, rust fungi of the genus Puccinia constitute a taxonomically large group with high phytopathological risk, comprising over 6000 species [2]. The basidiomycete *Puccinia graminis* Pers. f. sp. *tritici* (*Pgt*), the causal agent of wheat stem rust, is one of the most destructive phytopathogens; it can destroy susceptible genotypes owing to rapid epiphytotic spread [3].

*Pgt* affects a wide range of cereals, including bread and durum wheat, triticale, and barley, and is widespread globally: Europe [4,5,6,7,8], Asia and the Middle East [9], Australia, and North America [10,11]. Favorable climatic conditions make *Pgt* particularly threatening in many African countries with developed agricultural sectors [12,13]. In Russia, stem rust is recorded annually and causes the greatest damage in the Volga region and Western Siberia, with focal outbreaks in the North Caucasus [14,15,16,17,18].

The pathogen is dispersed by aerial transport of urediniospores, which are the summer asexual spores of the fungus, and produces multiple generations during the growing season. Upon germination in surface water or dew, the spores form germ tubes that penetrate the host tissue through stomata. The developing pustules on stems rupture the epidermis, increase transpiration, reduce the photosynthetic area and disrupt water balance, leading to decreased plant productivity. Yield losses caused by *Pgt* can reach 70% or more [19]. Moreover, data from 2023 indicate that over 80% of global wheat cultivars are susceptible under natural and artificial inoculation conditions [6,9,20,21].

Current control measures against the causal agent of stem rust include breeding resistant cultivars, fungicidal control, surveillance and detection of infection foci, and the establishment of working groups and national/international programs (including forecasting and predictive breeding). However, new highly virulent races are reported annually, such as Ug99 (TTKSK), which originated in East Africa and is capable of transcontinental spread with substantial impacts on agricultural economies [4].

In this context, international monitoring programs of *Pgt* genetic diversity have become increasingly important, including the Borlaug Global Rust Initiative (BGRI) and the Global Rust Reference Center (GRRC), which provide isolate phenotyping, population-structure analysis, and identification of *Sr* genes for marker-assisted selection (MAS) of resistant cultivars [22,23]. Fungal pathogens, ubiquitous components of biological diversity, are controlled by fungicides in agricultural systems, but this control is associated with an increasing risk of resistance development [24].

Among commercially available fungicides with high activity against *Pgt* are Alto Super (active ingredients: propiconazole and cyproconazole), Amistar Extra (active ingredients: azoxystrobin and cyproconazole) and Tilt (active ingredient: propiconazole) (Syngenta, www.syngenta.com). According to FRAC, of the 46 fungicide classes, only three are effective against rust fungi (FRAC, www.frac.info): quinone outside inhibitors (QoIs), demethylation inhibitors (DMIs) (affect the *Pgt* gene *Cyp51*) and succinate dehydrogenase inhibitors (SDHIs) [25].

Although rust fungi (order *Pucciniales*) are generally considered at low risk of resistance development, single-nucleotide polymorphisms (SNPs) can reduce target affinity [26], and thus serve as molecular indicators of *Pgt* population heterogeneity and as quantitative markers of intrapopulation variability for predictive breeding [27].

Analysis of the molecular genetic diversity of *Pgt* is of fundamental importance for tracking intrapopulation changes, identifying new sources of resistance, and developing predictive breeding strategies [28]. Genotyping of *Pgt* populations is essential for monitoring the evolution of virulent races (e.g., Ug99 and TTKSK-derived subgroups); this enables prediction of the erosion of *Sr*-gene effectiveness (e.g., *Sr24*, *Sr31*, *SrTmp*) and timely adjustment of breeding programs, thereby helping to reduce crop losses [22].

Standard approaches for assessing *Pgt* genetic diversity include phenotyping virulence on sets of differential lines, phylogenetic analysis, and molecular differentiation at the species and population levels using SSR and SNP markers in conserved regions such as *ITS* and *EF1-α* [29,30]. Under conditions of intense fungicide pressure, genotyping of fungicide-target genes (*SdhA–SdhD*, *Cyp51*) becomes particularly pertinent, since SNPs can reduce pathogen sensitivity to chemical control [24,26].

Modern *Pgt* genotyping methods typically rely on costly sequencing of genome-wide libraries to detect multiple SNPs and indels at target loci. Previously, we developed an economical, high-throughput targeted amplicon sequencing protocol based on Oxford Nanopore Technologies (ONT-TAS), demonstrating its effectiveness on plant genomes (sunflower and wheat) with an analysis time of less than 4.5 h and complete haplotype reconstruction for assessment of allelic diversity in germplasm collections [31,32]. Although Illumina short read sequencing offers higher per-base accuracy for SNP calling, ONT TAS provides a cost-effective and time-efficient alternative for large-scale population screening, enabling near-real-time monitoring of *Pgt* populations. However, the molecular diversity of fungicide-target genes in Russian *Pgt* populations remains largely unexplored. Here, we report the first adaptation of ONT TAS to basidiomycetes, specifically to *Pgt*, to quantify intrapopulation variability in virulence and fungicide-target genes. We hypothesized that populations from regions with intensive fungicide use would exhibit higher frequencies of non-synonymous SNPs in target genes (*SdhA–SdhD* and *Cyp51*) compared to low-pressure areas. This work addresses an existing gap by providing the first comprehensive molecular genetic characterization of Russian *Pgt* populations, thereby enabling predictive breeding and global pathogen monitoring.

Regular monitoring of crop disease development is essential for the early detection of novel pathogen foci and for evaluating the efficacy of control measures, particularly in agriculturally significant regions. In 2024, a focus of *Pgt* infection on triticale was identified within the territory of the Main Botanical Garden of the Russian Academy of Sciences (Moscow Region, Russia), and a pathogen population was subsequently collected. For comparative purposes, a local *Pgt* population was collected in the Chelyabinsk Region during the same year. The study also included herbarium material of *Pgt* collected in 2023 in the Saratov Region, as no *Pgt* foci were detected in that area during route surveys conducted in 2024. This absence was likely attributable to seasonal weather conditions and the routine application of fungicides, both of which contributed to reduced infectious pressure. Expeditionary surveys carried out in the Volga region between 2023 and 2025 confirm the presence of multiple active *Pgt* foci on both spring and winter wheat in this intensive grain-producing area, where high fungicide usage is common. These findings underscore the phytosanitary significance of the issue [21,33].

The aim of this study was to conduct a comparative analysis of virulence and variability profiles of fungicide-target genes in *Pgt* populations from regions of Russia. For the first time in Russia, we performed a comprehensive molecular-genetic analysis of *Pgt* isolates from regions with contrasting fungicide pressure (Moscow Region, Volga Region, and Southern Urals) using targeted amplicon sequencing on the Oxford Nanopore Technologies (ONT-TAS) platform for high-resolution genotyping of allelic variants in key fungicide-susceptibility genes (*SdhA–SdhD*, *Cyp51*).

## 2. Materials and Methods

### 2.1. Sampling of Pgt

*Pgt* populations were collected in three regions of Russia: the Saratov, Chelyabinsk and Moscow regions, from breeding plots and experimental farms chosen to represent gradients in fungicide use, agricultural intensity, and climatic conditions.

Three regions were selected for sampling *Pgt* populations: Saratov (2023), Chelyabinsk (2024), and Moscow (2024). The choice of regions and sampling years was determined by material availability and the presence of active infection foci during field surveys. It should be noted, however, that this study design does not allow full control of all varying factors: region, harvest year, host genotype, and environmental conditions differ simultaneously. An ideal approach for comparative population analysis would involve collecting samples within a single growing season on a single host species using standardized protocols. However, this was not feasible in the present study due to logistical constraints and the episodic nature of the infection. Consequently, the observed differences between populations may reflect not only regional specificity of the pathogen but also the effects of harvest year, host genotype, and weather conditions, all of which are taken into account when interpreting the results.

The rationale for region selection was based on official open data for 2025 from the Federal State Statistics Service of the Russian Federation. The Saratov Region (2.1 million ha of grain) and the Chelyabinsk Region (1.2 million ha) are centers of intensive agricultural production characterized by high levels of fungicide use and climatic conditions favorable for *Pgt* development in some years. The Moscow Region is characterized by a relatively small but strategically important grain acreage (less than 150,000 ha), high urbanization, and minimal fungicide pressure, providing a natural infectious background for comparison with intensive production regions [34].

In the Saratov Region, samples were collected in 2023 from the susceptible spring durum wheat cultivar “Elizavetinskaya” at the Federal State Budgetary Scientific Organization “Federal Center of Agriculture Research of the South-East Region” (51.57547 N, 46.00422 E) [33]. The Chelyabinsk stem rust population was collected in 2024 from the spring bread wheat cultivar “Duet” from breeding plots at the State scientific institution Chelyabinsk scientific-research institute of agriculture of the Russian Academy of Agricultural Sciences (54.928277 N, 60.758201 E). In the Moscow Region, *Pgt* populations were collected in 2024 from winter triticale cultivar “Tit” from breeding plots maintained by the Department of Wide Crosses at the Main Botanical Garden of the Russian Academy of Sciences (55.858736 N, 37.032041 E).

**Figure 1:**
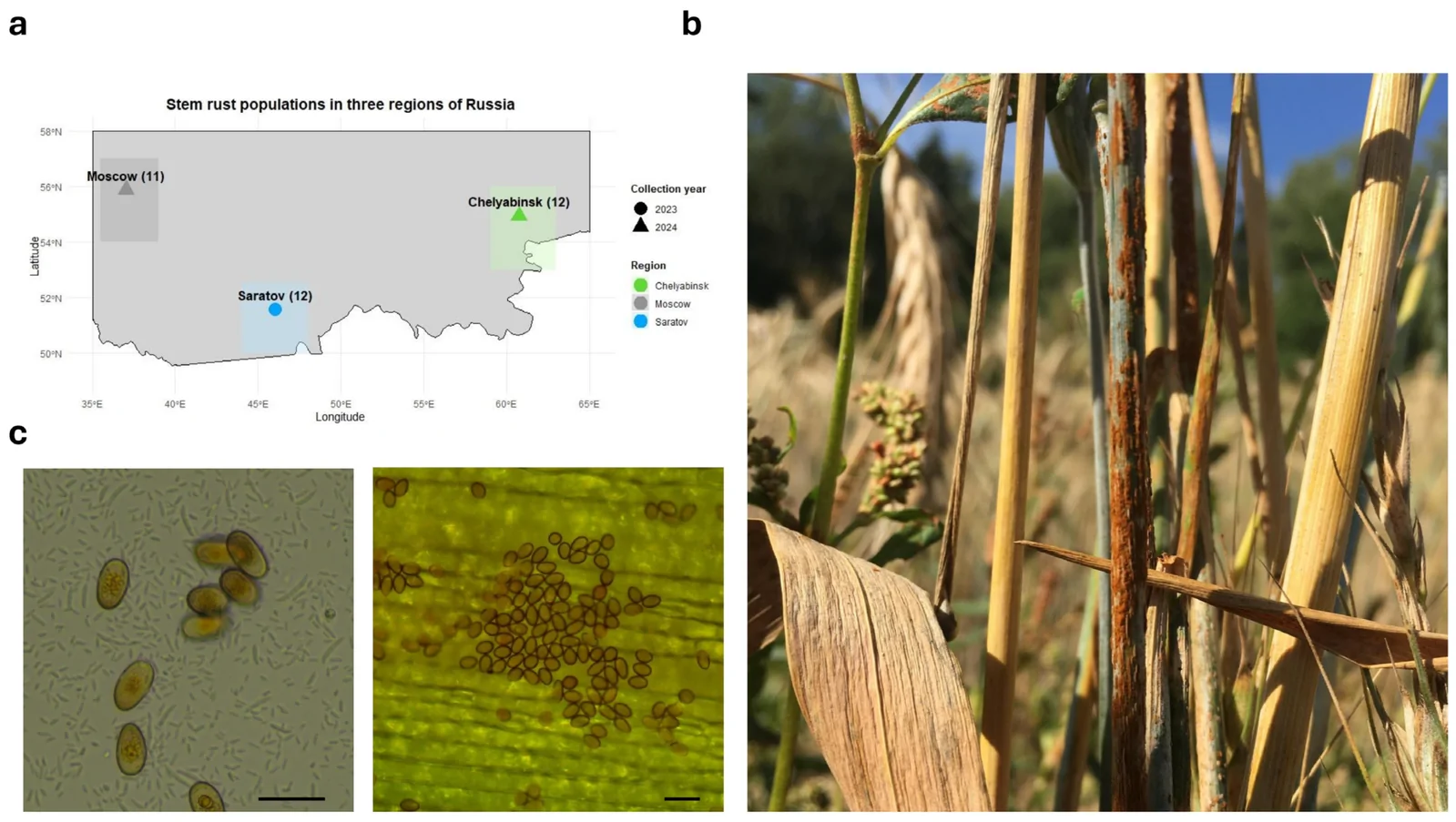
Collection sites of *Pgt* populations used in this study. (**a**) Symptoms of *Pgt* detected on genetically diverse varieties of winter wheat and triticale at field sites in the Moscow, Saratov, and Chelyabinsk regions (the X-axis represents geographical longitude, and the Y-axis represents latitude). Bars indicate the number of monopustular isolates obtained from each population and analyzed at each location. (**b**) Representative image of the orange uredinia characteristic of stem rust lesions observed on wheat stems at the sampling sites. (**c**) Urediniospores isolated from infected wheat plants (magnifications ×100 and ×400). Scale bar represents 50 µm.

For each year of pathogen material collection, differences in temperature and moisture conditions were observed, which influenced pathogen development. From each population, we obtained monopustular isolates of the fungus by single-pustule transfers on susceptible wheat plants: 12 from the Saratov population, 12 from the Chelyabinsk population, and 11 from the Moscow population (Figure 1).

### 2.2. Morphological Identification and Visualization of Pgt

Primary confirmation of stem rust was performed by cytological examination of infected wheat stems bearing characteristic orange pustules using a Carl Zeiss Primo Star microscope (Carl Zeiss Microscopy GmbH, Jena, Germany). Urediniospores were gently scraped from pustules with a sterile needle, suspended in sterile water on a microscope slide, and photographed at magnifications of ×100 and ×400 to document diagnostic morphological features (Figure 1c).

### 2.3. Inoculation and Reproduction of Urediniospore Isolates

Spore propagation followed a standard protocol [35]. Urediniospore suspensions were prepared at a concentration of 1 mg urediniospores per 1 mL of sterile water with the addition of Tween 80. Seedlings of the susceptible cultivar Khakasskaya were inoculated at the seedling stage. Inoculated plants were incubated in the dark for 16 h at 23 °C and ≥90% relative humidity to promote spore germination, after which they were transferred to a 16/8 h light/dark photoperiod at 23 °C for disease development and pustule formation. Single pustules were selected for subsequent molecular analysis to ensure the genetic purity of the isolates.

### 2.4. Phenotypic Assessment of Virulence

To propagate *P. graminis* populations and obtain monopustular isolates, we used a method widely adopted in international practice on seedlings under controlled temperature and humidity conditions [35]. The virulence profiles of the populations were determined on a set of differential lines (North American differential set: *Sr5*, *Sr21*, *Sr9e*, *Sr7b*, *Sr11*, *Sr6*, *Sr8a*, *Sr9g*, *Sr36*, *Sr9b*, *Sr30*, *Sr17*, *Sr9a*, *Sr9b*, *Sr10*, *SrTmp*, *Sr24*, *Sr31*, *Sr38*, *SrMcN*) and additional lines with *Sr* genes: *Sr2compl*, *Sr8b*, *Sr11*, *Sr12*, *Sr13*, *Sr15*, *Sr17+13*, *Sr20*, *Sr22*, *Sr25*, *Sr26*, *Sr27*, *Sr28*, *Sr29*, *Sr32*, *Sr33+5*, *Sr33*, *Sr35*, *Sr36*, *Sr37*, *Sr39*, *Sr40*, *Sr44*, *SrDb*, *Sr WLD*, *SrWLD-1*, *Sr24+31*, *Sr36+31*, *Sr24+31*, *Sr31+36*, *Sr24+36*, *Sr2+23*, *Sr7a+12*, *Sr7b+18*, *Sr26+9g*. The cultivar Aurora (carrying the *Sr31* gene) and the cultivars Khakasskaya and Morocco (susceptible controls) were also included in the analysis. Leaf inoculation was performed using the method described above, with three replicates.

The wheat seedling response to inoculation with a suspension of stem rust urediniospores was assessed 10–12 days after inoculation using the standard Stakman scale [36]. Resistance or susceptibility of a genotype was determined based on reaction types observed across three replicates. Plants showing reaction types “0”, “0;”, “1”, and “2” were classified as resistant, while reaction types “3”, “4”, and “X” were classified as susceptible.

### 2.5. DNA Extraction, PCR Amplification, and Phylogenetic Analysis of Pgt

To initially confirm that the pathogen populations collected from wheat and triticale belong to the specialized form *Puccinia graminis* f. sp. *tritici*, an analysis was performed using genetic markers targeting the internal transcribed spacer (ITS) region and the elongation factor 1-alpha (*EF1-α*) gene. For this purpose, monopustular isolates were obtained from each stem rust pathogen population: 12 isolates from the Saratov population, 12 isolates from the Chelyabinsk population, and 11 isolates from the Moscow population. DNA was extracted from the *Pgt* isolates using a “CytoSorb” reagent kit (Syntol, Moscow, Russia) with modifications. The modifications consisted of adding 1 g of washed, autoclaved fine sand to the protocol during the sample homogenization step to improve disruption of the fungal chitinous cell wall. DNA quality was assessed using a NanoDrop One microvolume spectrophotometer (Thermo Fisher Scientific, Waltham, MA, USA). The same DNA preparations were used in subsequent analyses of fungicide-target genes via nanopore sequencing (Section 2.6).

Standard genetic markers targeting the internal transcribed spacer (*ITS*) region and the elongation factor 1-alpha (*EF1*-*α*) gene were used for phylogenetic analysis. The PCR program was identical for all primer pairs (Supplementary Table S1). PCR reactions (50 µL) contained 1 µL genomic DNA (3–5 ng), 10 pM of each primer, 10 µL ScreenMix (5X) (Eurogen, Moscow, Russia), and nuclease-free water to a final volume of 50 µL. PCR products were separated by electrophoresis on a 1.2% agarose gel. The horizontal cameras of the Sub-Gell GT system and the PowerPac HC power supply unit (BioRad, Berkeley, CA, USA) were used for the analysis.

PCR products were visualized using a GelDoc XR+ imaging system (Bio-Rad, Hercules, CA, USA). Bands corresponding to target amplicons were excised from the gel using an ECX-M transilluminator (Vilber Lourmat, Eberhardzell, Germany). Amplicons were purified with the CleanUp Mini kit (Eurogen, Russia) and Sanger-sequenced by Syntol (Russia). Resulting nucleotide sequences were queried against the NCBI database using BLAST (version BLAST+ 2.17.0) to identify homologous sequences.

Sequence alignment was performed using the ClustalW algorithm. To minimize the effect of incomplete terminal regions on phylogenetic analysis, the final alignment was manually trimmed to the length of the overlapping fragments. Subsequent analyses, including calculation of evolutionary distances using the p-distance model [37] and tree construction by the Neighbor-Joining method [38], were performed in MEGA12 [39] using the pairwise deletion option to retain all available data.

### 2.6. SNP Screening of Fungicide-Target Genes in Pgt Using ONT-TAS

The rationale for SNP screening of fungicide-target genes (*SdhA–SdhD*, *Cyp51*) in monopustular *Pgt* isolates is based on phenotyping results for populations from the Saratov, Chelyabinsk, and Moscow regions, which revealed variability in the effectiveness of Sr genes among the differential lines and additional *Sr* lines. Because the regional virulence profile of pathogen populations determines crop protection strategies and fungicide pressure, we applied targeted amplicon sequencing (ONT-TAS) using the Rapid Barcoding kit on the Oxford Nanopore Technologies platform for precise genotyping of polymorphisms.

The first step after extracting genomic DNA from single-pustule isolates of the stem rust pathogen (Section 2.5) was PCR amplification of fungicide-target genes: *SdhA*, *SdhB*, *SdhC*, *SdhD*, and *Cyp51*. For PCR amplification, we used primers described by Savva, Bryan et al. [40]. In addition, we designed specific molecular markers for each gene using Primer3.0 (accessed 1 November 2025). Primers were designed exclusively within the coding sequences (CDS) of each gene, and multiple primer-pair options were evaluated per gene (Supplementary Table S2). To maximize coverage of the target regions, primers were placed as close as possible to the 5′ and 3′ ends of the coding sequences and were required to encompass all known single-nucleotide polymorphisms (SNPs) associated with fungicide resistance.

The concentration and integrity of purified amplicons were assessed using a NanoDrop One UV–visible spectrophotometer (Thermo Scientific, Waltham, MA, USA) and a Qubit 4 fluorometer with Qubit dsDNA BR Assay Kits (Thermo Fisher Scientific, Waltham, MA, USA), respectively, and were additionally verified by agarose gel electrophoresis. PCR product concentrations were normalized prior to sequencing.

### 2.7. ONT-TAS Library Preparation and Sequencing of Target Amplicons

In this experiment, we analyzed 35 pooled samples, each containing 5 amplicons for the genes *Cyp51*, *SdhA*, *SdhB*, *SdhC*, and *SdhD*. For the rapid barcoding kit (Oxford Nanopore Technologies, Oxford, UK), each sample was mixed with 9 μL of the pooling PCR product (50 ng) and 1 μL of one rapid barcode. The mixture was incubated at 30 °C for 2 min followed by incubation at 80 °C for 2 min. All barcoded DNA samples were pooled, and 350 μL of pooled DNA was mixed with an equal volume of SynMag Beads (Syntol, Moscow, Russia). After 5 min of incubation at room temperature on a Hula mixer, the barcoded DNA was cleaned twice with 80% ethanol and eluted with 12 μL of Elution Buffer (EB). Incubation was performed at 37 °C for 10 min. An aliquot of the barcoded DNA was used to obtain a total volume of 11 μL with EB. In a fresh LoBind tube, dilute 0.5 uL Rapid Adapter (RA) and 1.17 uL Adapter Buffer (ADB). One microliter of Adapter mix was added to the barcoded DNA, and the mixture was incubated at room temperature for 10 min. Then, 12 µL barcoded amplicons were mixed with 37.5 µL Sequencing Buffer II (SBII) and 25.5 µL Loading Beads II (LBII). Sequencing was performed using MinION and the flow cell SQK-LSK114. Basecalling was performed by Guppy (Version 6.3.8).

The obtained reads were aligned to the reference sequences (ID sequences: *SdhA*—NW_003526578.1:c641794-638437; *SdhB*—KAA1124466.1; *SdhC*—XM_003320603.2; *SdhD*—KAA1134160; *Cyp51*—OR359784.1).

In total, 175 PCR products were purified and mixed together to obtain final samples for barcoding (Supplementary Figure S1) and sequencing using a single MinION flow cell. The analysis of read generation during the sequencing procedure showed that read coverage of 50×–250× was obtained for each amplicon of an individual accession after three hours of sequencing. In total, we obtained ~38,290 reads with an N50 value of around 1.06 kb (Oscore > 8). On average, there were about 1000 reads associated with each barcode.

### 2.8. SNP Calling

Nanopore reads, obtained during sequencing, were aligned according to each barcode using minimap2 with the “-map-ont” argument for aligning noisy long reads of ~10% error rate to a reference genome [41]. We added headers to mapped reads and converted sam to bam followed by bam file sorting using SAMtools [42]. When using Samtools, arguments were applied to exclude secondary, chimeric, and failed alignments while retaining only reads with mapping Qscore ≥ 30. These filtering limitations were sufficient for reliable SNP identification, despite challenges posed by the genetic complexity of monopustular isolates of the stem rust pathogen, which hindered analysis with standard bioinformatic tools. Sequence analysis generated mapped reads, revealing coverage depths of 50× to 250× for each amplicon across individual accessions, which enabled reliable detection of structural variations. Then SNPs and InDels were identified, and the corresponding allele identifiers were assigned if they were previously known and deposited in GenBank. SNPs in exons were identified from high-coverage data and validated by BLAST alignment against known reference sequences, including those present in ≥50% of reads with the same barcode.

## 3. Results

### 3.1. Phenotypic Assessment of Virulence

In this study, we analyzed three *Pgt* populations collected from areas with differing fungicide pressure. We identified effective *Sr* genes (Supplementary Table S3).

**Figure 2:**
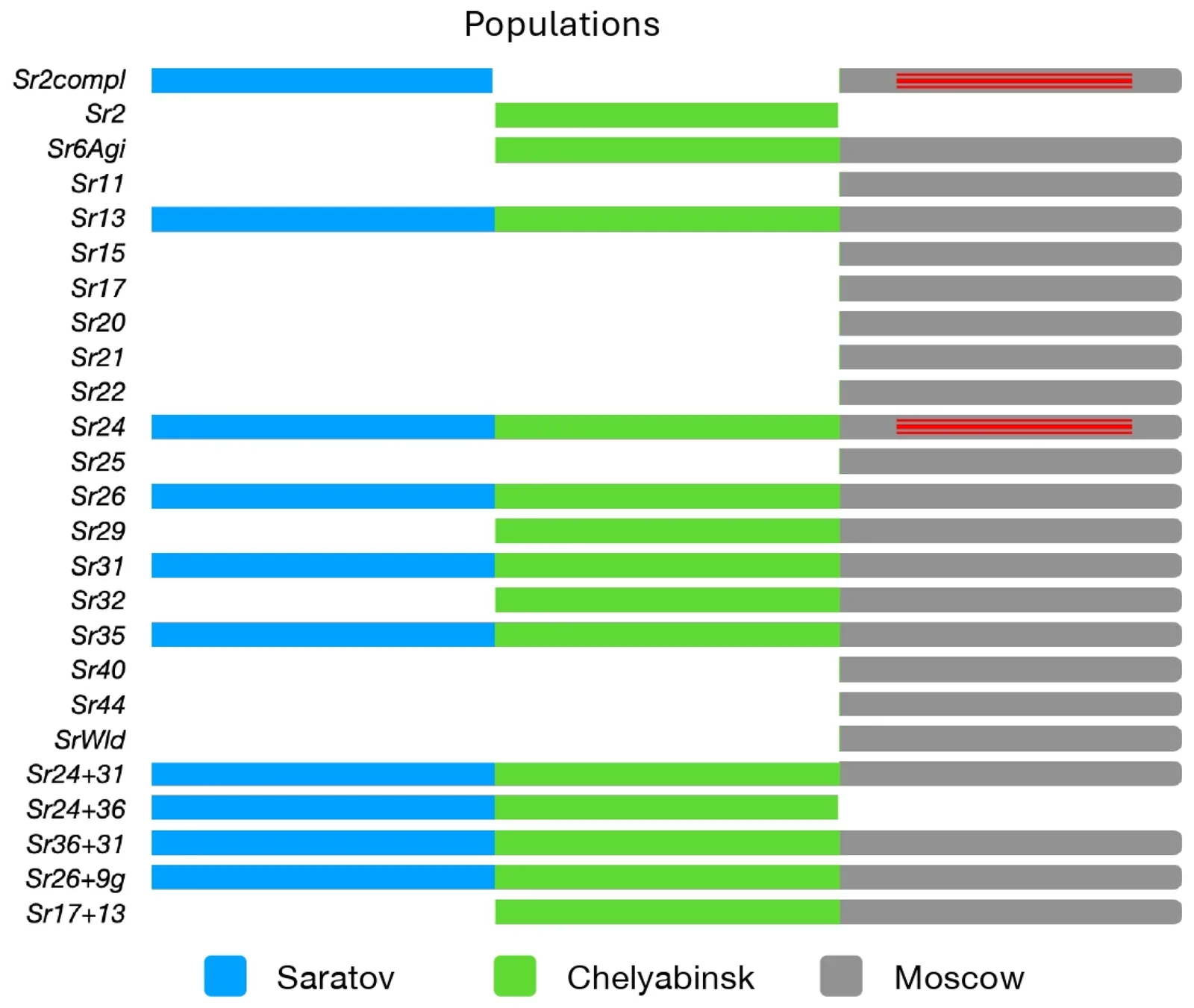
Effective stem rust (*Sr*) resistance genes in relation to *Pgt* populations collected from Saratov (blue), Chelyabinsk (green), and Moscow (gray) regions. The color coding corresponds to the geographic origin of each population. The genes *Sr2compl* and *Sr24*, indicated by red hatching, fail to provide resistance against the Moscow *Pgt* population.

Data on population virulence assessed at the seedling stage on differential varieties and additional lines are presented in Figure 2. The *Sr* genes *Sr13*, *Sr26*, *Sr31*, *Sr32*, and *Sr35*, as well as the gene combinations *Sr24+31*, *Sr36+31*, *Sr26+9g*, and *Sr17+13*, were effective against all analyzed fungal populations (Figure 2).

For the Saratov *Pgt* population, the following genes were effective: *Sr2compl*, *Sr13*, *Sr24*, *Sr26*, *Sr31*, *Sr35*, and the gene combinations *Sr24+31*, *Sr24+36*, *Sr36+31*, and *Sr26+9g*. For the Chelyabinsk population, effective genes included *Sr2*, *Sr13*, *Sr24*, *Sr26*, *Sr29*, *Sr31*, *Sr32*, *Sr35*, and the combination *Sr26+9g; Sr6Agi* was also effective, as were the combinations *Sr24+31*, *Sr36+31*, *Sr24+36*, and *Sr17+13*.

Considering the Moscow population separately, the following genes and combinations were effective: *Sr11*, *Sr13*, *Sr15*, *Sr17*, *Sr20*, *Sr21*, *Sr22*, *Sr25*, *Sr26*, *Sr29*, *Sr31*, *Sr32*, *Sr35*, *Sr40*, *Sr44*, *SrWld*, *Sr24+31*, *Sr36+31*, *Sr26+9g*, *Sr17+13*, and *Sr6Agi.* However, the Moscow population was virulent on lines carrying *Sr2compl* and *Sr24*.

It should be noted that *Pgt* populations from different regions exhibit substantial differences. Virulence screening of these populations indicates that genetic resistance is beginning to erode regionally, manifested by loss of effectiveness of Sr genes that are commonly deployed in breeding programs (for example, *Sr2*, *Sr24*, and *Sr25*). Consequently, fungicide applications will remain necessary to maintain a favorable phytosanitary situation, but they also raise concerns about the potential development of *Pgt* resistance to chemical control.

### 3.2. Phylogenetic Analysis of Pgt Isolates

**Figure 3:**
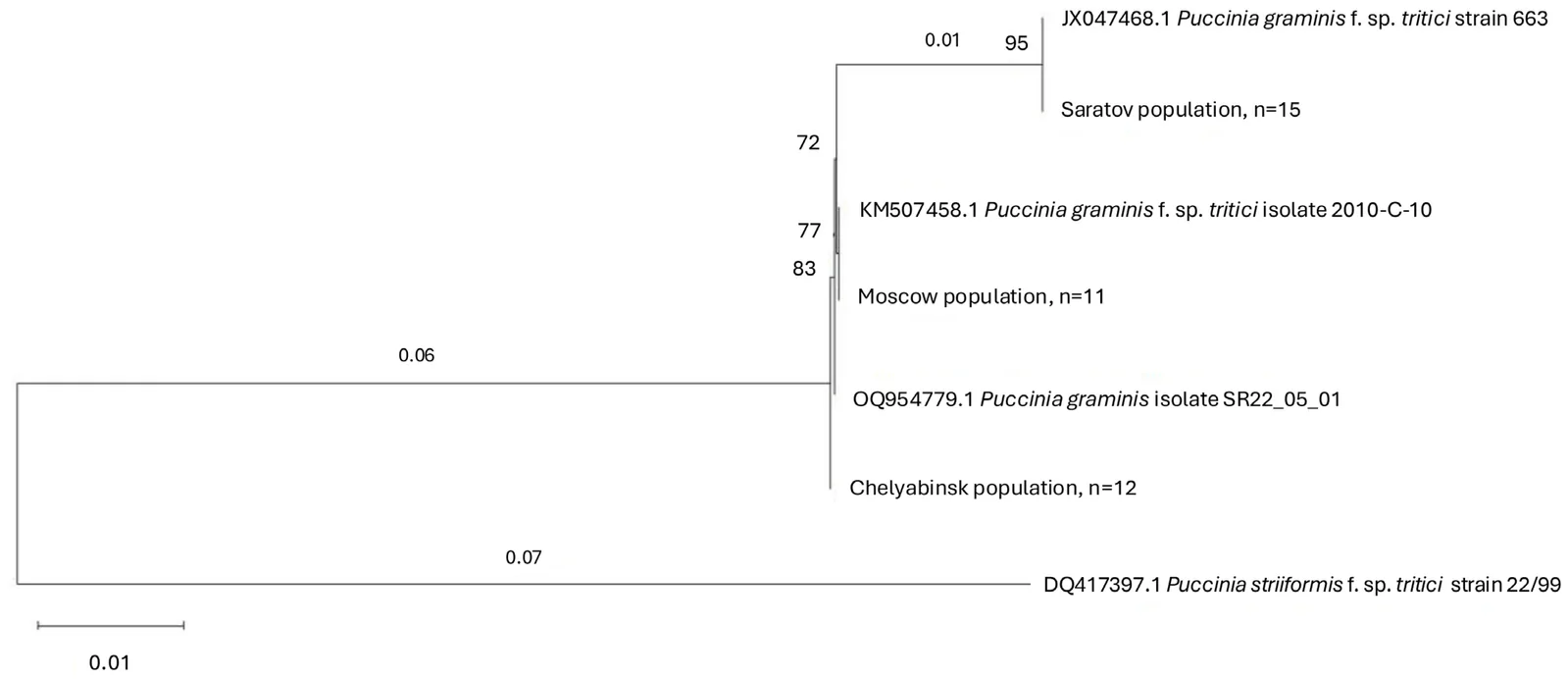
Phylogenetic tree of single-pustule *Pgt* isolates from the Saratov, Chelyabinsk, and Moscow populations, reconstructed from internal transcribed spacer (*ITS*) sequences. All three populations (Saratov, Moscow, Chelyabinsk) cluster within a single *P. graminis* f. sp. *tritici* clade. *P. striiformis* f. sp. *tritici* was used as the outgroup.

**Figure 4:**
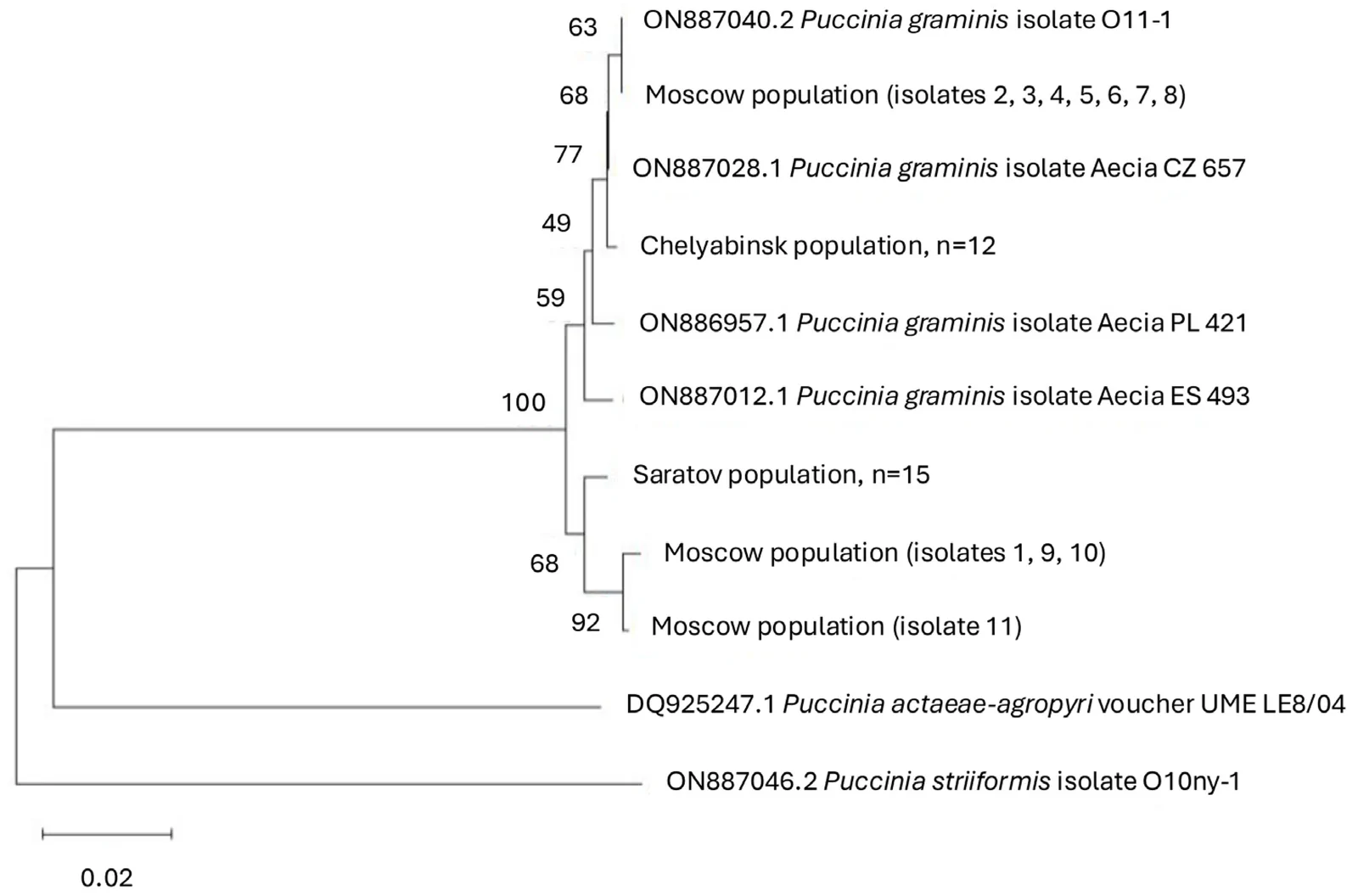
Phylogenetic tree of single-pustule *Pgt* isolates from the Saratov, Chelyabinsk, and Moscow populations, reconstructed from elongation factor (*EF1*-*α*) gene sequences. The Saratov population shows a close genetic relationship with a subset of Moscow isolates. The Moscow sample exhibits clear intrapopulation structure. The Chelyabinsk population clusters within the same clade but shows minor genetic differentiation.

Phylogenetic analysis confirmed that the populations collected from wheat and triticale belong to the wheat-specialized formae speciales—*Puccinia graminis* f. sp. *tritici*. Standard genetic markers—the internal transcribed spacer (*ITS*) (Figure 3) and the elongation factor (*EF1-α*) (Figure 4)—were used for the analysis. DNA extracted from single-pustule isolates of the three *Pgt* populations served as the input matrix, and *Puccinia striiformis* f. sp. *tritici* was used as the outgroup.

The evolutionary history was inferred using the Neighbor-Joining method [38]. The optimal tree with the sum of branch lengths = 0.141 is shown. The percentage of replicate trees in which the associated taxa clustered together in the bootstrap test (1000 replicates) is shown next to the branches [43]. The tree is drawn to scale, with branch lengths (above the branches) in the same units as those of the evolutionary distances used to infer the phylogenetic tree. The evolutionary distances were computed using the p-distance method [37] and are in the units of the number of base differences per site. The analytical procedure encompassed 7 nucleotide sequences. The pairwise deletion option was applied to all ambiguous positions for each sequence pair, resulting in a final data set comprising 214 positions.

The optimal tree with the sum of branch lengths = 0.304 is shown. The analytical procedure encompassed 11 nucleotide sequences. The pairwise deletion option was applied to all ambiguous positions for each sequence pair, resulting in a final data set comprising 527 positions.

Phylogenetic analysis using the internal transcribed spacer (*ITS*) marker confirmed that all single-pustule isolates from the Saratov, Chelyabinsk, and Moscow populations belong to *Pgt* (Figure 3). Based on elongation factor 1-alpha (*EF1*-*α*) sequences, isolates from the Saratov and Chelyabinsk populations form a single cluster with complete intrapopulation identity, whereas Moscow isolates comprise several haplotypes showing high homology (98–100%) to GenBank reference sequences (Figure 4; Table 1).

### 3.3. Targeted Amplicon Sequencing by ONT-TAS

Targeted amplicon sequencing (ONT-TAS) on the Oxford Nanopore platform was used to detect intrapopulation SNPs in fungicide-target genes (*Cyp51*, *SdhA*, *SdhB*, *SdhC*, *SdhD*) in 35 single-pustule *Pgt* isolates. Specific primers were designed to flank the coding sequences for PCR amplification of each gene. Despite high-quality DNA preparations (Qubit; QIAamp DNA Mini Kit extraction), we did not obtain amplicons of *Pgt* isolates from the Moscow population for the *SdhA* and *Cyp51* genes (Table 2), likely owing to high GC content and/or stable secondary structures in the target regions.

We analyzed 35 pooled samples, generating a total of 175 PCR products (five per isolate). Amplicons were purified, pooled, barcoded, and sequenced on a single MinION flow cell. In ~3 h, ~38,290 reads were obtained (Oscore > 8, N50 = 1.06 kb). Filtered reads were used to construct consensus haplotypes and investigate allelic variants.

### 3.4. SNP Validation

Reads obtained from ONT sequencing were aligned to fasta files of reference gene sequences, with failed reads filtered out. This approach enabled the identification of allelic variants in five rust susceptibility genes through comparison with NCBI reference sequences. Given sufficient sequencing depth across all five analyzed genes, we identified alleles that differed from known variants. The obtained BAM files contained SNPs in the *SdhA*, *SdhB*, *SdhC*, *SdhD*, and *Cyp51* genes of the analyzed *Pgt* samples. The distribution of synonymous, non-synonymous, and nonsense mutations across genes and populations is presented in the Supplementary Materials (Supplementary Table S4).

**Figure 5:**
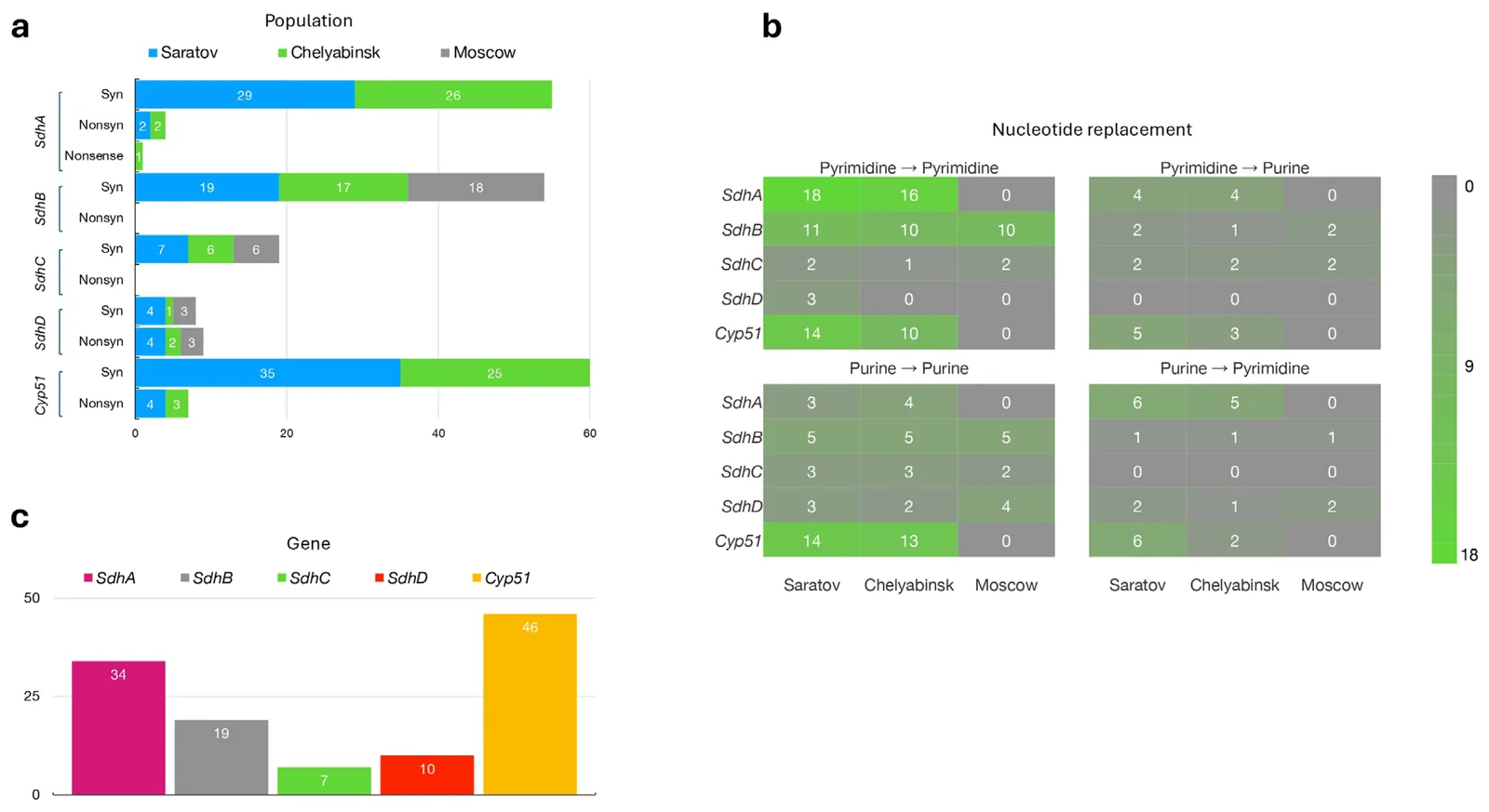
Single-nucleotide substitutions in fungicide-target genes identified in single-pustule *Pgt* isolates. (**a**) Number of SNPs in single-pustule *Pgt* isolates by region of pathogen collection. (**b**) Characterization of SNPs in *Pgt* clones by region of collection. (**c**) Total number of substitutions for each fungicide-target gene.

Our analysis revealed that the majority of identified SNPs resulted in synonymous amino-acid substitutions (Figure 5). However, a proportion of non-synonymous single-nucleotide substitutions was also observed, and a single nonsense mutation introducing a premature stop codon in the *SdhA* gene (position 580, C → T substitution) was detected in one isolate from the Chelyabinsk population. Notably, this mutation was identified at the DNA level; its functional impact on protein function and cell viability was not assessed in the present study and requires experimental verification.

In the Chelyabinsk population, the *SdhA* gene contained 26 synonymous substitutions, 2 non-synonymous substitutions, and 1 nonsense mutation; *SdhB* contained 17 synonymous substitutions; *SdhC* contained 6 synonymous substitutions; *SdhD* contained 1 synonymous and 2 non-synonymous substitutions; and *Cyp51* contained 25 synonymous and 3 non-synonymous substitutions.

In the Moscow population, polymorphisms were detected as follows: *SdhB*—18 synonymous substitutions; *SdhC*—6 synonymous substitutions; *SdhD*—3 synonymous and 3 non-synonymous substitutions.

In the Saratov population, *SdhA* carried 29 synonymous and 2 non-synonymous substitutions; *SdhB*—19 synonymous substitutions; *SdhC*—7 synonymous substitutions; *SdhD*—4 synonymous and 4 non-synonymous substitutions; and *Cyp51*—35 synonymous and 4 non-synonymous substitutions (Figure 5a).

We observed that certain non-synonymous mutation positions recurred within the same gene groups among clones from the Saratov and Chelyabinsk populations. In the *SdhA* gene, recurrent non-synonymous nucleotide substitutions were detected: at nucleotide position 1459, a T → C substitution resulted in a Ser → Pro amino-acid change, and at position 1835, a substitution resulted in a Leu → Ser change. A similar pattern was observed for *Cyp51* at positions 683 (Asn → Ser), 1432 (Asn → Asp), and 1447 (Pro → Ala; corresponding to amino-acid position 483) (Table 3). No recurrent non-synonymous SNPs or nonsense mutations were detected among *Pgt* clones in the *SdhB*, *SdhC*, or *SdhD* genes.

We detected a high frequency of transitions—that is, substitutions that do not change the purine–pyrimidine orientation within a nucleotide pair. In the *Cyp51* gene, purine → purine and pyrimidine → pyrimidine substitutions were most frequent, with up to 14 such substitutions per isolate across the three populations. Overall, the most common mutation types were pyrimidine—pyrimidine substitutions. In *SdhA*, Saratov single-pustule isolates exhibited the highest number of these substitutions (18), while Chelyabinsk isolates showed 16. Between 10 and 14 pyrimidine transitions were observed across all studied fungicide-target genes in the three *Pgt* populations (Figure 5b).

We also tallied the total number of SNPs per gene. *Cyp51* and *SdhA* exhibited the highest SNP counts among the genes studied. *SdhA* showed the greatest incidence of both transversions (four-to-six-point substitutions) and transitions (up to 18 substitutions). By contrast, *SdhC* and *SdhD* were more conserved, with 7 and 10 single-nucleotide substitutions detected, respectively (Figure 5c).

## 4. Discussion

The phenotypic and genotypic data obtained are generally consistent with the hypothesis of a fungicide-associated selection gradient that may affect the structure of *Pgt* populations in the studied regions of the Russian Federation. The Moscow population, sampled under conditions of lower fungicidal pressure and a short growing season, was characterized by high haplotype diversity at the *Ef1a* locus and virulence polymorphism, presumably resulting from mitotic recombination of urediniospores. In contrast, the Saratov and Chelyabinsk populations, which experienced intense fungicidal pressure (SDHIs and azoles), exhibited greater clonal homogeneity and the presence of fixed non-synonymous variants in target genes.

However, interpretation of these results requires consideration of several methodological limitations that preclude unambiguous attribution of the observed differences to any single factor. In the present study, the following variables varied simultaneously:

(1) Year of sample collection: The Saratov population was collected in 2023, whereas the Chelyabinsk and Moscow populations were collected in 2024; this may reflect interannual climatic differences affecting pathogen development and population structure.
(2) Region and associated climatic conditions: Temperature regimes, precipitation patterns, and growing season duration vary significantly between the Volga region, the Southern Urals, and the Moscow region, influencing pathogen population dynamics.
(3) Host genotype and species: Samples were collected from different varieties and species: spring durum wheat (Saratov), spring common wheat (Chelyabinsk), and winter triticale (Moscow). Differences in host resistance and susceptibility could have affected population composition.
(4) Fungicidal pressure intensity: Although fungicidal pressure was estimated to be high in the Saratov and Chelyabinsk regions and low in the Moscow region, it was not quantified and may vary depending on the year and agricultural practices.
(5) Environmental conditions: Temperature, humidity, and other abiotic factors varied across collection years and could have influenced infection development and isolate survival.

Thus, the study design does not allow separation of the effects of geographical factors, fungicide use, and other simultaneously varying variables. Accordingly, all conclusions regarding fungicide-associated selection are preliminary and require confirmation in controlled experiments, including sampling within a single growing season, the use of isogenic host lines, and quantitative assessment of fungicidal pressure. Despite these limitations, the present study represents a first step in assessing the molecular diversity of *Pgt* populations in Russian regions with contrasting agroecological conditions and provides a basis for further research with a more rigorous design.

The molecular organization of target genes provides a biological basis for their differential variability. Succinate dehydrogenase (complex II) is a four-subunit enzyme complex (*SdhA–SdhD*) that represents the smallest complex in the respiratory chain, transferring electrons from succinate to the ubiquinone pool. Mutations in the *Sdh*-genes have previously been repeatedly associated with reduced sensitivity to SDHI fungicides in phytopathogenic fungi [44,45]. We observed that the studied *SdhA–SdhD* loci are characterized by a high GC content and are subject to C/T transitions.

In the present study, a nonsense mutation in the *SdhA* gene (position 580, C → T substitution) was identified in a single isolate from the Chelyabinsk population. This mutation introduces a premature stop codon in the coding sequence. Since *SdhA* encodes the flavoprotein subunit of succinate dehydrogenase (mitochondrial complex II), such a variant may severely impair respiratory function and cell viability. However, no direct fungicide sensitivity tests or respiratory activity assays were conducted in this work. Therefore, this mutation cannot be interpreted as a confirmed resistance determinant or a functionally validated polymorphism.

Furthermore, *Pgt* urediniospores are dikaryotic; thus, a nonsense mutation present in only one nuclear copy may be phenotypically masked by the wild-type allele through complementation. Additionally, although sequencing data were of high quality and filtered by read depth, the possibility of a sequencing artefact cannot be fully excluded. At this stage, we consider the detected nonsense variant as a candidate polymorphism requiring further validation using culture-based phenotypic assays and protein-level analyses. If functionally relevant, its maintenance in the population could potentially be explained by heterokaryosis, compensatory mutations, or a lack of selective pressure against this locus under the current agricultural practices.

Recurrent mutations found in different populations, such as *SdhA* 1459 T/C, should be regarded as candidate alleles for altered sensitivity, as potentially spreading variants within populations, or as a result of convergent adaptation under SDHI selection pressure, but not as confirmed determinants of reduced susceptibility without phenotypic confirmation. Similarly, the nonsense mutation in *SdhA* (position 580) identified in this study should be regarded as a candidate variant only, and not as a confirmed marker of reduced fungicide sensitivity, until functional validation is performed.

According to the literature, most confirmed mutations conferring reduced sensitivity to SDHI fungicides are localized in the *SdhB*, *SdhC*, and *SdhD* subunits, which form the quinone-binding pocket of mitochondrial complex II. In particular, documented substitutions at conserved positions have been reported in *Zymoseptoria tritici*, *Botrytis cinerea*, *Pyrenophora teres*, and *Corynespora cassiicola*, including *SdhB*-H267Y/L, *SdhB*-P225L, *SdhC*-N75S, *SdhD*-D129E, and others [46,47,48,49,50]. These mutations have been validated phenotypically.

In contrast, the non-synonymous substitutions in the *SdhA* gene identified in our study (positions 1459 and 1835, resulting in Ser → Pro and Leu → Ser substitutions, respectively), as well as the nonsense mutation at position 580, do not coincide with well-characterized resistance “hot spots” documented in the literature. It should be noted that mutations in the *SdhA* subunit, which encodes a flavoprotein, are less frequently associated with SDHI resistance compared with mutations in *SdhB*, *SdhC*, and *SdhD*. Moreover, the presence of a non-synonymous substitution in any of the *Sdh*-genes does not in itself constitute evidence of reduced fungicide sensitivity, as the functional significance of a substitution depends on its localization within the protein structure, its effect on ligand binding, and potential compensatory mechanisms.

In contrast, *Cyp51*—a membrane cytochrome P450 enzyme with a sterically complex active center—predominantly accumulates conservative substitutions that do not disrupt the spatial structure of the protein. Therefore, a similarly cautious approach is necessary when interpreting variations in *Cyp51*. In the present study, non-synonymous substitutions in *Cyp51* were identified at positions 683, 1432, and 1447, and these variants may be associated with altered sensitivity to azoles. However, the absence of direct sensitivity tests precludes their interpretation as confirmed resistance markers. At this stage, they can only be regarded as molecular candidates requiring functional validation.

Accordingly, we do not assume functional equivalence between the identified substitutions and known resistance mutations. At this stage, all detected non-synonymous variants, including those in *SdhA* and *Cyp51*, are considered exclusively as molecular candidates requiring phenotypic validation.

Modern Russian wheat and triticale cultivars are characterized by a scarcity of *Sr2* and *Sr24* and by a narrow spectrum of effective *Sr* genes, predominantly *Sr31* in both winter and spring forms and *Sr25* and *Sr6Agi* in spring cultivars [51,52]. Such genetic uniformity increases the risk of epiphytotics following the emergence of virulent races. The recurrence of SNPs at *SdhA* 1459 T → C and *Cyp51* 1432 A → G in agriculturally important regions supports selective enrichment of *Pgt* populations with alleles potentially associated with reduced fungicide sensitivity. However, alternative population-genetic explanations should not be excluded in addition to the hypothesis of fungicide-associated selection.

First, repeated variants may be a consequence of common origin. If the studied *Pgt* populations share a common ancestor or originated from the same source population, then similar mutations may be present in different regions due to hereditary variability rather than independent adaptation. This is especially likely for panmictic populations of rust fungi, which are characterized by a high degree of genetic connectivity over long distances.

Second, standing genetic variability (neutral polymorphisms) may explain the presence of variants unrelated to selective advantage. Many SNPs in fungicide-target genes can be neutral or nearly neutral, particularly if they are localized outside functionally significant protein domains.

Third, gene flow between populations (migration) is a particularly likely explanation for *Pgt*, given the ability of urediniospores to undergo long-range aerogenic transport over hundreds or thousands of kilometers. Thus, the similar mutations identified in the Saratov and Chelyabinsk populations may result from spore migration from a common source rather than independent adaptation to fungicides in each region.

Despite these alternative explanations, we favor the hypothesis of fungicide-associated selection for the following reasons. First, the mutations we identified are localized in fungicide-target genes (*SdhA* and *Cyp51*), which are under direct selective pressure when SDHI and DMI fungicides are applied. Second, these mutations were found specifically in regions with high agricultural production intensity and regular fungicide use (Saratov and Chelyabinsk regions), whereas in the Moscow region, with minimal fungicidal pressure, such repeated non-synonymous substitutions were not detected. Third, the pattern of mutation distribution corresponds to what is expected under local adaptation rather than random drift or migration.

The virulence data obtained indicate differential effectiveness of several *Sr*-genes across the studied populations. For all analyzed populations, *Sr13*, *Sr26*, *Sr31*, *Sr32*, and *Sr35*, and the gene combinations *Sr24+31*, *Sr36+31*, *Sr26+9g*, and *Sr17+13* remained effective. These results are generally consistent with recent Russian studies. Volkova et al. (2024) reported the absolute effectiveness of *Sr31* under field conditions in the Southern region of Russia, whereas the effectiveness of *Sr24* and *Sr25* varied depending on the year and environmental conditions [53]. In the study by Baranova et al. (2023), varieties from the Volga region also confirmed high effectiveness of *Sr31* (identified in 19 out of 126 varieties), whereas *Sr24* was found in only one variety, highlighting limited genetic diversity [21]. Skolotneva et al. (2026) demonstrated continued effectiveness of *Sr24* and *Sr31* regardless of pathogen origin (common or durum wheat) in the Altai region but noted that populations from different regions differ significantly in virulence [54].

Comparison with international data also confirms regional specificity. Jin et al. (2007) showed that the genes *Sr13*, *Sr22*, *Sr24*, *Sr25*, *Sr26*, *Sr27*, *Sr28*, *Sr32*, *Sr33*, *Sr35*, *Sr36*, *Sr37*, *Sr39*, *Sr40*, *Sr44*, *SrTmp*, and *SrTt-3* are effective against race TTKS (Ug99) at both the seedling and adult plant stages [35]. In China, Wu et al. (2020) reported that *Sr9e*, *Sr17*, *Sr21*, *Sr22*, *Sr26*, *Sr30*, *Sr31*, *Sr33*, *Sr35*, *Sr36*, *Sr37*, *Sr38*, *Sr47*, *SrTmp*, and *SrTt3* were effective against new *Pgt* races, whereas *Sr24* and *Sr28* were fully susceptible [55].

Particular attention should be paid to *Sr24*, which was effective against the Saratov and Chelyabinsk populations in our study but ineffective against the Moscow population. This indicates the existence of regional racial differences that must be taken into account in breeding programs. At the same time, Volkova et al. (2024) classified *Sr24* as moderately effective (10 MR–30 MR) under field conditions in Southern Russia, confirming the variability of its effectiveness [53].

It is important to note that the evaluation of *Sr*-gene effectiveness at the seedling stage, conducted in this study, represents a standard approach for primary screening but has limitations. As demonstrated by Jin et al. (2008) and Volkova et al. (2024), adult plant resistance (APR) could differ significantly from seedling-stage reactions [28,53]. Some genes can provide effective protection in the field while being ineffective at the seedling stage, and vice versa. This highlights the need for field trials to definitively assess the suitability of *Sr*-genes in breeding programs.

Collectively, our data indicate significant differences in virulence structure among the *Pgt* populations studied. This creates prerequisites for epiphytotics when new virulent races emerge, emphasizing the need for continuous monitoring of pathogen populations and expansion of the range of *Sr*-genes utilized.

Thus, the results of this study indicate differences among *Pgt* populations sampled in the Saratov, Chelyabinsk, and Moscow regions, both in virulence and in molecular variability within fungicide-target genes. The repeated detection of SNPs in *SdhA* and *Cyp51* in agronomically significant regions is consistent with possible selective enrichment of alleles potentially associated with adaptation to fungicides. However, this interpretation remains preliminary until confirmed by phenotypic tests. Furthermore, the reduced effectiveness of several *Sr*-genes, including *Sr2*, *Sr24*, and *Sr25*, underscores the need for continued monitoring of both pathogen populations and the spectrum of effective resistance genes in the studied regions. Therefore, the SNPs identified in the target genes *SdhA*– *SdhD* and *Cyp51* can serve as molecular markers for monitoring pathogen populations for use in early warning systems and for assessing the risk of resistance emergence under specific environmental conditions. Furthermore, the *Sr*-genes, the effectiveness of which was confirmed in this study, are strong candidates for marker-assisted selection (MAS) in breeding programs for resistant wheat and triticale varieties. Knowledge of regional virulence patterns enables the adaptation of fungicide management strategies, including the rotation of fungicides with different modes of action, to prevent the emergence of resistant strains in regions where non-synonymous substitutions in *SdhA* and *Cyp51* have been repeatedly identified. Overall, this work represents one of the first studies of *Pgt* in these regions based on targeted amplicon sequencing and provides a foundation for further research combining molecular analysis with biological validation of the identified variants.

## Figures and Tables

**Table 1:** Characterization of *Pgt* isolates by phylogenetic analysis.

Population Collection Region	Isolate Number	Type	Host	Year	% *EF1-α* Match	ID GenBank Ref *EF1-α*
Saratov	1–15	*Pgt*	Wheat	2023	98.62	ON887012.1
Chelyabinsk	1–12	*Pgt*	Wheat	2024	99.8	ON887028.1
Moscow	1, 9, 10	*Pgt*	Triticale	2024	98.62	ON886957.1
	2–8	*Pgt*			99.8	ON887040.2
	11	*Pgt*			98.62	ON887012.1

**Table 2:** Number of isolates used for ONT-TAS targeted amplicon sequencing.

Population Collection Region	Fungicide-Target Genes				
	*SdhA*	*SdhB*	*SdhC*	*SdhD*	*Cyp51*
Saratov	12	12	12	12	12
Chelyabinsk	9	12	11	11	11
Moscow	-	10	9	9	-

**Table 3:** Nonsense mutation and recurrent nonsynonymous SNP sites in clones from the Saratov and Chelyabinsk populations.

Gene	Isolate	Position of Nucleotide	Nucleotide Change	Amino Acid	Amino-Acid Position	Type of Replacement
*SdhA*	Chelyabinsk	580	C/T	R/stop	194	Nonsense
*SdhA*	Saratov	1459	T/C	S/P	487	Non-synonymous
		1835	T/C	L/S	612	
	Chelyabinsk	1459	T/C	S/P	487	
		1835	T/C	L/S	612	
*Cyp51*	Saratov	683	A/G	N/S	228	
		1432	A/G	N/D	478	
		1447	A/G	P/A	483	
	Chelyabinsk	683	A/G	N/S	228	
		1432	A/G	N/D	478	
		1447	A/G	P/A	483	

## Data Availability

The nanopore Targeted Amplicon Sequencing (ONT-TAS) data generated in this study are available in the NCBI Sequence Read Archive (SRA) under BioProject accession number PRJNA1510898. The data from the phylogenetic analysis of *Puccinia graminis* f. sp. *tritici* based on the EF1-α locus are available in GenBank under the following accession numbers: SUB16392995 Pg_EF_Saratov PZ809435; SUB16392995 Pg_EF_Chelyabinsk PZ809436; SUB16392995 Pg_EF_Moscow_G3 PZ809437; SUB16392995 Pg_EF_Moscow_G1 PZ809438; SUB16392995 Pg_EF_Moscow_G2 PZ809439.The data from the phylogenetic analysis of *Puccinia graminis* f. sp. *tritici* based on the ITS locus are available in GenBank under the following accession numbers: SUB16392883 Pg_ITS_Saratov PZ804550; SUB16392883 Pg_ITS_Chelyabinsk PZ804551; SUB16392883 Pg_ITS_Moscow PZ804552.

## Supplementary Materials

The following supporting information can be downloaded at https://www.mdpi.com/article/10.3390/jof12080604/s1. Figure S1: Visualization of sequences in a genomic browser for five *P. graminis* genes responsible for resistance to fungicides (Saratov samples are shown as an example (labeled “TB_№” in the “Genes” schematic)); Table S1: Molecular markers used to determine the phylogeny of monopustular isolates *Puccinia graminis* f. sp. *tritici* (*Pgt*) [56,57]; Table S2: PCR markers used for the amplification of fungicide target genes in monopustular isolates *Pgt*; Table S3: Resistance of *Sr* wheat lines to populations *Pgt*; Table S4: Distribution of synonymous, non-synonymous, and nonsense mutations by genes and populations.

## Abbreviations

The following abbreviations are used in this manuscript:

*Pgt*	*Puccinia graminis* f. sp. *tritici*
ONT-TAS	Target amplicon sequencing approach using Oxford Nanopore Technologies
SDH	Succinate dehydrogenase
ITS	Internal transcribed spacer
