# Supplementary material for: Virulence Profiles and Fungicide-Target-Gene Variability in Puccinia graminis f. sp. tritici Populations from Russian Regions
Source: J Fungi (Basel). 2026 Aug 13;12(8):604. doi: 10.3390/jof12080604 (PMC13514130; doi:10.3390/jof12080604)
Supplement: Supplementary file 1 [file jof-12-00604-s001.zip › Supplementary_all/Supplementary Figure S1 New. Visualization of sequences in a genomic browser for five P.graminis genes responsible for resistance to fungicides..pdf]

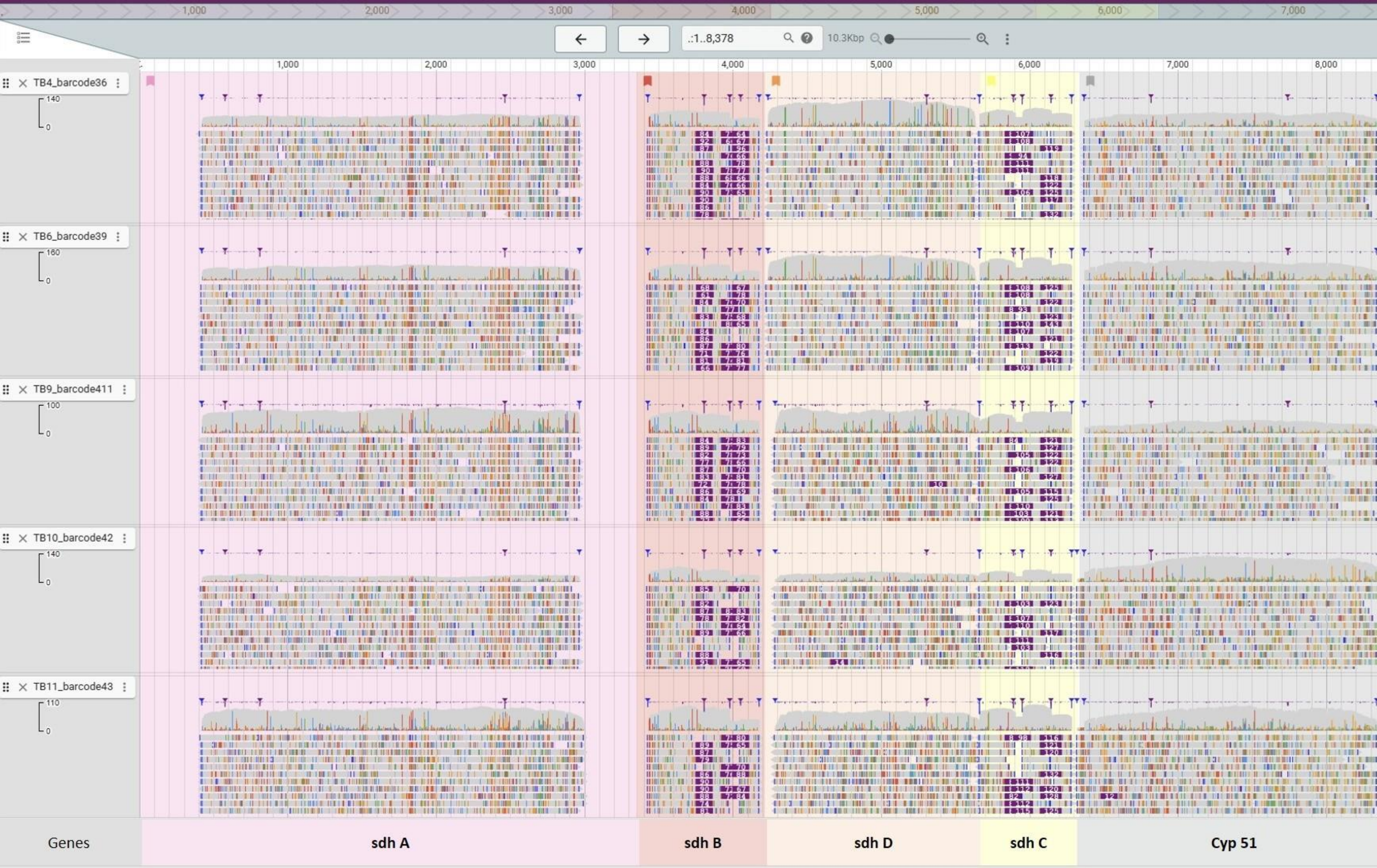

Supplementary Figure S1. Visualization of sequences in a genomic browser for five *P. graminis* genes responsible for resistance to fungicides *(Saratov samples are shown as an example (labeled "TB_№" in the "Genes" schematic))*
