# Supplementary material for: Virulence Profiles and Fungicide-Target-Gene Variability in Puccinia graminis f. sp. tritici Populations from Russian Regions
Source: J Fungi (Basel). 2026 Aug 13;12(8):604. doi: 10.3390/jof12080604 (PMC13514130; doi:10.3390/jof12080604)
Supplement: Supplementary file 1 [file jof-12-00604-s001.zip › Supplementary_all/Supplementary Table S1, S2, S3.pdf]

Supplementary Table S1. Molecular markers used to determine the phylogeny of monopustular isolates *Puccinia graminis* f. sp. *tritici (Pgt)*

Marker	Primers 5'-3'	Reference
ITS1rustF10d	TGAACCTGCAGAAGGATCATTA	[56]
ITS1rustR3c	TGAGAGCCTAGAGATCCATTGTTA	
EFbasidF	GTGCGGTGGTATTATCGACAAGC	[57]
EFbasidR	CATGTTGTCACCGTGCCATCC	
PCR conditions:		
95 °C – 180s	95 °C – 20s, 60 °C – 20s, 72 °C – 20s	72 °C – 300s
	35 cycles	

Supplementary Table S2. PCR markers used for the amplification of fungicide target genes in monopustular isolates *Pgt*

Gene	Marker	Primers 5’-3’	Reference
Cyp51	CYP51pgt-F	TCTTCCCTCATC GAC CCACT	This study
	CYP51pgt-R	GACCAACATAGTCTATCATCACA	
	CYP51pgtV2-R	CGAGTGGGTGTT CCTCCTAG	
	Cyp51v3F	CCCAAGAGTCAACAACACCG	[40]
	Cyp51v3R	AGTAAGTCGGTTTGCGAGGG	
SdhB	SdhBpgt-F	AACCATCCAGAACCTACTCA	This study
	SdhBpgt-R	CAGTAGCCATTTCTCTCTTGA	
SdhC	SdhCpgt-F	GATCATCCCGCCTGTCATTG	
	SdhCpgt-R	TCGAAGGAGAAATTAATGATGGC	
	SdhCv3F	CACAATGGCCACCACTTGTC	
	SdhCv3R	CAACACCGATCTCAAGAAAAGC	
SdhD	SdhDpgt-F	TCACTGAAGAGACCCGGCT	
	SdhDpgt-R	AGCCTTCCATGCCTTCTTGA	
	SdhDsT-F	TGGCTCTGTGACATGTGGAG	[40]
SdhA	SdhApgt-F	ACCCTAGTCTTCAAGCCACA	This study
	SdhApgt-R	GGA TGGACGGATGATTGAGG	
	SdhApgtV2-F	GTACGTTTGAACGGAGGAAC	
	SdhApgtV2-R	CCA AGGCCACCTCACTACTC	
	SdhAv3F	ACTTTTGCGAGCTTCAGACTTAG	[40]
	SdhAv3R	CTCACAGCCAATCAGCTTGC	
PCR conditions:			
95 °C – 300s		95 °C – 15s, 59 °C (57 °C for *SdhB*; 60 °C for *SdhC*, *SdhD*) – 15s, 72 °C – 100s	72 °C – 300s
		40 cycles	

Supplementary Table S3. Resistance of *Sr* wheat lines to populations *Pgt*

Lines with the Sr-gene	Resistance (type of reaction)		
	The Saratov *Pgt* population of 2023	The Chelyabinsk *Pgt* population of 2024	The Moscow *Pgt* population of 2024
Sr2 compl	1	2	3
Sr5	3	3	3
Sr6	3+	3	4
Sr7b	3	3	3
Sr8a	4	3	3+
Sr8b	4	3	3
Sr9a	4	3	3
Sr9b	3	3+	3
Sr9d	3	4	3
Sr9e	3	3	3
Sr9g	3	3	4
Sr10	3+	3+	3
Sr11	4	3-	1
Sr12	3	3	3
Sr13	1	1	0;
Sr15	3	3	0
Sr17	3	3	1
Sr20	3	4	2
Sr21	3	3	2
Sr22	3	3	1
Sr24	0;1	1	3+
Sr25	3	3	2
Sr26	;	1	0;
Sr28	3+	3	4
Sr29	3	0;	0;
Sr30	4	3	3
Sr31	1	2	0;
Sr32	3-	2	0;
Sr35	1	1	0;
Sr36	3	3	3
Sr38	4	3	4
Sr39	3	3	4
Sr40	3	3	0
Sr44	3	3	0
SrWld	3	3	1
SrTmp	4	4	4
SrMcN	4	4	4

Sr24+31	1	1	1
Sr36+31	1	1	1
Sr24+36	1	1;	3
Sr26+9g	1+	1	1
Sr7a+12	3	3	3
Sr7b+18	3	3	3
Sr17+13	1	0;	0;
Sr6Agi	3	1	0;
