# Supplementary material for: Virulence Profiles and Fungicide-Target-Gene Variability in Puccinia graminis f. sp. tritici Populations from Russian Regions
Source: J Fungi (Basel). 2026 Aug 13;12(8):604. doi: 10.3390/jof12080604 (PMC13514130; doi:10.3390/jof12080604)
Supplement: Supplementary file 1 [file jof-12-00604-s001.zip › Supplementary_all/Supplementary Table S4. Distribution of synonymous, non-synonymous, and nonsense mutations by genes and populations.pdf]

Supplementary Table S4. Distribution of synonymous, non-synonymous, and nonsense mutations by genes and populations

Gene	Isolates of the population	Position of nucleotide	Nucleotide replacement	Type of replacement	Amino acid	Amino acid position
SdhA	Saratov	86	C/G	Synonymous	S/S	29
		117	C/T	Synonymous	P/P	39
		225	T/C	Synonymous	A/A	75
		255	C/T	Synonymous	N/N	85
		315	G/T	Synonymous	G/G	105
		375	C/T	Synonymous	T/T	125
		396	T/C	Synonymous	L/L	132
		468	G/A	Synonymous	G/G	156
		567	C/G	Synonymous	A/A	189
		600	G/C	Synonymous	L/L	200
		624	G/A	Synonymous	L/L	208
		678	T/C	Synonymous	D/D	226
		723	T/C	Synonymous	D/D	241
		729	C/T	Synonymous	T/T	243
		747	A/C	Synonymous	A/A	249
		945	T/C	Synonymous	G/G	315
		1188	C/T	Synonymous	T/T	396
		1233	A/T	Synonymous	G/G	411
		1341	T/G	Synonymous	S/S/	447
		1459	T/C	Non-synonymous	S/P	487
		1512	C/T	Synonymous	A/A	504
		1626	C/T	Synonymous	H/H	542
		1632	T/C	Synonymous	V/V	544
		1635	C/T	Synonymous	V/V	545
		1638	C/T	Synonymous	D/D	546
		1641	C/G	Synonymous	S/S	547
		1659	A/G	Synonymous	V/V	553
		1662	T/C	Synonymous	T/T	554
		1731	A/T	Synonymous	A/A	557
		1740	G/A	Synonymous	T/T	580
		1835	T/C	Non-synonymous	L/S	612
	Chelyabinsk	86	C/G	Synonymous	S/S	29
		117	C/T	Synonymous	P/P	39
		204	C/T	Synonymous	A/A	68
		207	T/A	Synonymous	G/G	69
		225	T/C	Synonymous	A/A	75
		255	C/T	Synonymous	N/N	85
		315	G/T	Synonymous	G/G	105
		375	C/T	Synonymous	T/T	125
		396	T/C	Synonymous	L/L	132
		468	G/A	Synonymous	G/G	156
		567	C/G	Synonymous	A/A	189
		580	C/T	Nonsense	R/*	194
		600	G/C	Synonymous	L/L	200
		624	G/A	Synonymous	L/L	208
		678	T/C	Synonymous	D/D	226
		723	T/C	Synonymous	D/D	241
		729	C/T	Synonymous	T/T	243
		747	A/C	Synonymous	A/A	249
		1188	C/T	Synonymous	T/T	396
		1233	A/T	Synonymous	G/G	411
		1459	T/C	Non-synonymous	S/P	487
		1635	C/T	Synonymous	V/V	545
		1638	C/T	Synonymous	D/D	546
		1641	C/G	Synonymous	S/S	547
		1659	A/G	Synonymous	V/V	553
		1662	T/C	Synonymous	T/T	554
		1731	A/T	Synonymous	A/A	557
		1740	G/A	Synonymous	T/T	580
		1835	T/C	Non-synonymous	L/S	612

SdhB	Chelyabinsk	78	C/T	Synonymous	S/S	26
		81	A/T	Synonymous	I/I	27
		99	C/T	Synonymous	S/S	33
		105	C/G	Synonymous	S/S	35
		117	T/C	Synonymous	A/A	39
		126	C/T	Synonymous	A/A	42
		138	T/C	Synonymous	A/A	46
		216	G/A	Synonymous	K/K	72
		225	A/G	Synonymous	L/L	75
		345	T/C	Synonymous	C/C	115
		366	C/T	Synonymous	I/I	122
		399	C/T	Synonymous	R/R	133
		408	A/G	Synonymous	K/K	136
		420	G/A	Synonymous	A/A	140
		663	T/C	Synonymous	S/S	221
		669	T/C	Synonymous	Y/Y	223
		711	G/A	Synonymous	G/G	237
	Moscow	78	C/T	Synonymous	S/S	26
		81	A/T	Synonymous	I/I	27
		105	C/G	Synonymous	S/S	35
		117	T/C	Synonymous	A/A	39
		126	C/T	Synonymous	A/A	42
		138	T/C	Synonymous	A/A	46
		138	T/A	Synonymous	A/A	46
		216	G/A	Synonymous	K/K	72
		225	A/G	Synonymous	L/L	75
		345	T/C	Synonymous	C/C	115
		366	C/T	Synonymous	I/I	122
		399	C/T	Synonymous	R/R	133
		408	A/G	Synonymous	K/K	136
		420	G/A	Synonymous	A/A	140
		528	C/T	Synonymous	N/N	176
		663	T/C	Synonymous	S/S	221
		669	T/C	Synonymous	Y/Y	223
		711	G/A	Synonymous	G/G	237
	Saratov	78	C/T	Synonymous	S/S	26
		81	A/T	Synonymous	I/I	27
		99	C/T	Synonymous	S/S	33
		105	C/G	Synonymous	S/S	35
		117	T/C	Synonymous	A/A	39
		126	C/T	Synonymous	A/A	42
		138	T/C	Synonymous	A/A	46
		138	T/A	Synonymous	A/A	46
		216	G/A	Synonymous	K/K	72
		225	A/G	Synonymous	L/L	75
		345	T/C	Synonymous	C/C	115
		366	C/T	Synonymous	I/I	122
		399	C/T	Synonymous	R/R	133
		408	A/G	Synonymous	K/K	136
		420	G/A	Synonymous	A/A	140
		528	C/T	Synonymous	N/N	176
		663	T/C	Synonymous	S/S	221
		669	T/C	Synonymous	Y/Y	223
		711	G/A	Synonymous	G/G	237
SdhC	Saratov	93	C/T	Synonymous	P/P	31
		270	G/A	Synonymous	R/R	90
		306	G/A	Synonymous	A/A	102
		312	C/A	Synonymous	A/A	104
		315	G/A	Synonymous	L/L	105
		366	C/T	Synonymous	V/V	122
		537	T/A	Synonymous	T/T	179
	Chelyabinsk	93	C/T	Synonymous	P/P	31
		270	G/A	Synonymous	R/R	90
		306	G/A	Synonymous	A/A	102
		312	C/A	Synonymous	A/A	104
		315	G/A	Synonymous	L/L	105
		537	T/A	Synonymous	T/T	179
	Moscow	93	C/T	Synonymous	P/P	31
		270	G/A	Synonymous	R/R	90
		312	C/A	Synonymous	A/A	104
		315	G/A	Synonymous	L/L	105
		366	C/T	Synonymous	V/V	122
		537	T/A	Synonymous	T/T	179

SdhD	Saratov	14	G/C	Non-synonymous	R/T	5
		56	A/G	Synonymous	D/D	19
		65	G/A	Non-synonymous	S/N	22
		154	T/C	Non-synonymous	S/P	52
		193	G/T	Non-synonymous	A/S	65
		348	C/T	Synonymous	H/H	116
		411	C/T	Synonymous	A/A	137
		600	A/G	Synonymous	Q/Q	200
	Chelyabinsk	56	A/G	Synonymous	D/D	19
		65	G/A	Non-synonymous	S/N	22
		193	G/T	Non-synonymous	A/S	65
Cyp51	Saratov	73	T/C	Non-synonymous	F/S	18
		84	G/C	Synonymous	S/S	28
		198	C/T	Synonymous	Y/Y	66
		213	C/T	Synonymous	Y/Y	71
		240	A/G	Synonymous	K/K	80
		276	A/G	Synonymous	K/K	92
		354	A/G	Synonymous	E/E	118
		363	A/T	Synonymous	T/T	121
		366	A/C	Synonymous	A/A	122
		381	G/A	Synonymous	V/V	127
		525	T/C	Synonymous	G/G	175
		654	G/C	Synonymous	A/A	218
		683	A/G	Non-synonymous	N/S	228
		690	C/T	Synonymous	I/I	230
		693	G/A	Synonymous	Q/Q	231
		720	C/T	Synonymous	G/G	240
		774	G/A	Synonymous	R/R	258
		792	G/A	Synonymous	E/E	264
		798	T/C	Synonymous	A/A	266
		804	C/T	Synonymous	I/I	268
		810	T/C	Synonymous	I/I	270
		813	C/A	Synonymous	A/A	271
		816	T/C	Synonymous	L/L	272
		867	C/A	Synonymous	L/L	289
		870	C/T	Synonymous	H/H	290
		939	A/G	Synonymous	G/G	313
		963	C/T	Synonymous	D/D	321
		981	T/C	Synonymous	R/R	327
		984	G/A	Synonymous	V/V	328
		993	C/A	Synonymous	P/P	331
		1038	C/A	Synonymous	P/P	346
		1179	A/C	Synonymous	A/A	393
		1179	A/G	Synonymous	A/A	393
		1290	A/G	Synonymous	G/G	430
		1290	A/C	Synonymous	G/G	430
		1380	C/G	Synonymous	L/L	460
		1386	T/C	Synonymous	T/T	462
		1432	A/G	Non-synonymous	N/D	478
		1447	A/G	Non-synonymous	P/A	483
	Chelyabinsk	84	G/C	Synonymous	S/S	28
		198	C/T	Synonymous	Y/Y	66
		240	A/G	Synonymous	K/K	80
		276	A/G	Synonymous	K/K	92
		354	A/G	Synonymous	E/E	118
		363	A/T	Synonymous	T/T	121
		381	G/A	Synonymous	V/V	127
		683	A/G	Non-synonymous	N/S	228
		690	C/T	Synonymous	I/I	230
		693	G/A	Synonymous	Q/Q	231
		720	C/T	Synonymous	G/G	240
		792	G/A	Synonymous	E/E	264
		798	T/C	Synonymous	A/A	266
		804	C/T	Synonymous	I/I	268
		810	T/C	Synonymous	I/I	270
		867	C/A	Synonymous	L/L	289
		870	C/T	Synonymous	H/H	290
		939	A/G	Synonymous	G/G	313
		963	C/T	Synonymous	D/D	321
		981	T/C	Synonymous	R/R	327
		984	G/A	Synonymous	V/V	328
		993	C/A	Synonymous	P/P	331
		1179	A/G	Synonymous	A/A	393
		1290	A/G	Synonymous	G/G	430
		1380	C/G	Synonymous	L/L	460
		1386	T/C	Synonymous	T/T	462
		1432	A/G	Non-synonymous	N/D	478
		1447	A/G	Non-synonymous	P/A	483
